# Supplementary material for: Evaluation of Chronic Dietary Risk of Trifloxystrobin and Bupirimate in Cucumber Based on Supervised Residue Test
Source: Foods. 2025 May 14;14(10):1745. doi: 10.3390/foods14101745 (PMC12110765; doi:10.3390/foods14101745)
Supplement: Supplementary file 1 [file foods-14-01745-s001.zip › foods-3613839-supplementary.pdf]

Table S1. Fragmentation pattern of five pesticides in MRM mode

Compounds	Molecular Formula	Precursor Ion (m/z)	Product Ion (m/z)	Decluster Potential (eV)	Collision Energy (eV)
Trifloxystrobin	$C_{20}H_{19}F_3N_2O_4$	409.1	186.1*<br>145.0	40	23<br>63
Trifloxystrobin acid	$C_{19}H_{17}F_3N_2O_4$	395.0	186.0*<br>148.0	80	10<br>10
Bupirimate	$C_{13}H_{24}N_4O_3S$	317.2	166.1*<br>210.2	90	31<br>33
Ethirimol	$C_{11}H_{19}N_3O$	210.2	140.1*<br>98.1	100	29<br>33

* Quantifier

Table S2. Terminal residues of trifloxystrobin, trifloxystrobin (including trifloxystrobin acid), bupirimate and ethirimol during different intervals in cucumber

Compounds	Intervals (d)	Residues (mg/kg)	Supervised trials median residue (STMR, mg/kg)	Highest residue (HR, mg/kg)
Trifloxystrobin (including trifloxystrobin acid)	3	0.010 (15), 0.020 (2), 0.023, 0.024, 0.028, 0.029, 0.030, 0.031, 0.04	0.010	0.04
	5	＜0.010 (22), 0.020, 0.022	0.010	0.022
	7	＜0.010 (24)	0.010	0.01
Bupirimate	3	＜0.010 (21), 0.010, 0.025, 0.076	0.010	0.076
	5	＜0.010 (23), 0.011	0.010	0.011
	7	＜0.010 (24)	0.010	0.011
Ethirimol	3	＜0.010 (22), 0.22, 0.3	0.010	0.3
	5	＜0.010 (24), 0.12, 0.16	0.010	0.16
	7	＜0.010 (22), 0.035, 0.036	0.010	0.036

Table S3. Chronic dietary risk assessment of trifloxystrobin (including trifloxystrobin acid), bupirimate and ethirimol

Food Categories	Fi (kg)	Trifloxystrobin (including trifloxystrobin acid)				Bupirimate				Ethirimol			
		Reference residue limits (mg kg^{-1})	NEDI (mg)	ADI (mg)	RQ_c (%)	Reference residue limits (mg kg^{-1})	NEDI (mg)	ADI (mg)	RQ_c (%)	Reference residue limits (mg kg-1)	NEDI (mg)	ADI (mg)	RQ_c (%)
Rice (products)	0.2399	0.1 (China)	0.02399										
Flour (products)	0.1385	0.2 (China)	0.0277										
Other cereals	0.0233	0.02 (China)	0.000466										
Tubers	0.0495	0.2 (China)	0.0099										
Dried beans (products)	0.016												
Dark vegetables	0.0915	0.7 (China)	0.06405										
light vegetables	0.1837	0.01 (STMR)	0.001837			0.010 (STMR)	0.001837			0.010 (STMR)	0.001837		
Pickles	0.0103												
Fruits	0.0457	3 (China)	0.1371			0.5 (China)	0.02285			0.1 (China)	0.00457		
Nuts	0.0039	0.02 (China)	0.000078	ADI× 63				ADI× 63					
Livestock and poultry	0.0795												
Milk and dairy products	0.0263												
Egg and its products	0.0236												
Fish and shrimp	0.0301												
Vegetable oil	0.0327	0.01 (EU)	0.000327										
Animal oil	0.0087												
Sugar, starch	0.0044												
Salt	0.012	0.02 (US)	0.00024										
soy sauce	0.009	0.03 (CAC)	0.00027										
Total	1.0286		0.266	2.52	10.60%		0.0247	3.15	0.8		0.0064	2.205	0.3

Table S4. Chronic dietary exposure risks of trifloxystrobin (including trifloxystrobin acid), bupirimate and ethirimol to different Chinese populations in different age roups

Age Class	Gender	Chronic risk quotient (RQ$_C$, %)											
		Trifloxystrobin (including trifloxystrobin acid)				Bupirimate				Ethirimol			
0-35 months	Female	0.144	1.292	2.167	2.167	0.115	1.034	1.733	1.733	0.164	1.477	2.476	2.476
	Male	0.087	1.111	1.302	1.905	0.070	0.889	1.042	1.524	0.100	1.270	1.488	2.177
3-5 years	Female	0.109	0.762	1.150	1.270	0.087	0.610	0.920	1.016	0.124	0.871	1.315	1.452
	Male	0.094	0.893	1.174	1.947	0.075	0.714	0.939	1.558	0.107	1.020	1.341	2.225
6-14 years	Female	0.063	0.753	0.951	1.405	0.050	0.602	0.761	1.124	0.072	0.861	1.087	1.605
	Male	0.064	0.760	1.047	1.287	0.051	0.608	0.838	1.030	0.073	0.869	1.196	1.471
15-49 years	Female	0.041	0.491	0.683	0.895	0.032	0.393	0.546	0.716	0.046	0.561	0.780	1.023
	Male	0.038	0.458	0.616	0.822	0.030	0.366	0.493	0.657	0.043	0.523	0.704	0.939
50-74 years	Female	0.035	0.417	0.530	0.667	0.028	0.333	0.424	0.534	0.040	0.476	0.605	0.763
	Male	0.031	0.424	0.554	0.694	0.025	0.339	0.443	0.556	0.036	0.485	0.633	0.794
>75 years	Female	0.000	0.313	0.352	0.403	0.000	0.250	0.282	0.322	0.000	0.357	0.403	0.460
	Male	0.053	0.405	0.463	0.505	0.042	0.324	0.370	0.404	0.060	0.463	0.529	0.577
